# Steam Explosion Modified κ-Carrageenan Structure and Its Jelly Application

**DOI:** 10.3390/gels10120791

**Published:** 2024-12-03

**Authors:** Mengfan Lin, Qingyu Yang, Changrong Wang, Zebin Guo

**Affiliations:** 1 College of Food Science, Fujian Agriculture and Forestry University, Fuzhou 350002, China; lin_mengfan@163.com (M.L.); qingyu980206@163.com (Q.Y.); wcr9948@163.com (C.W.); 2 Integrated Scientific Research Base of Edible Fungi Processing and Comprehensive Utilization Technology, Ministry of Agriculture and Rural Affairs, Fuzhou 350002, China

**Keywords:** κ-carrageenan, steam explosion, jelly, structural properties

## Abstract

Steam explosion (SE) technology enhances the extraction efficiency of bioactive compounds and their physicochemical properties. This study compared the structural characteristics of κ-carrageenan extracted by SE-assisted alkali treatment and conventional alkali treatment from *Eucheuma*, as well as the quality attributes of the resulting jellies. The results indicate that SE treatment did not alter the species of carrageenan, but it significantly elevated the content of characteristic carrageenan groups, with the sulfate group content and 3,6-anhydrogalactose (3,6-AG) content increasing by 15.86% and 45.08%, respectively. The jellies prepared with κ-carrageenan following SE treatment demonstrated a more stable gel network structure, with an 80.9% increase in gel strength at a 1.5% κ-carrageenan concentration; the water precipitation rate of these jellies was minimized to 7.96 ± 0.69% when κ-carrageenan was added at a 1.7% concentration. These results suggest that SE treatment provides useful information for the application of κ-carrageenan in jelly.

## 1. Introduction

Seaweeds, one of the most abundant marine resources, are categorized into brown, green, and red algae based on their color. Within this group, red algae are the most abundant and are particularly rich in carrageenan, agar, and other polysaccharides [1], which are primarily utilized in industry for the production of hydrophilic colloids. *Eucheuma* is the most prevalent red algae in the world and serves as the principal source for hydrocolloid extraction due to its ease of cultivation, rapid growth, and high carrageenan content [2].

Carrageenan, also known as antlers algin or canageenin [3], is categorized into seven types based on the content and position of the sulfate groups in its structure. Among these, Kappa-carrageenan (κ-carrageenan), characterized by 25–30% of sulfate groups and 28–35% of dehydrated galactose, is the predominant type in industrial applications due to its superior gelation characteristics and rheological properties [4]. It exhibits broad utility across food, biomedical, and cosmetic industries, and is frequently employed as a thickening agent [5] or emulsifier [6].

**Figure 1:**
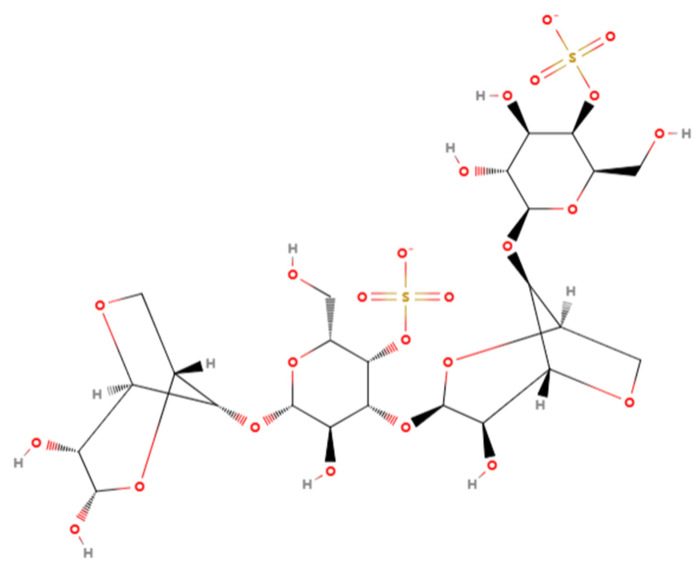
The chemical structure of κ-carrageenan.

The chemical structure of κ-carrageenan is shown in Figure 1. The κ-carrageenan obtained by boiling and alcohol precipitation after simple cleaning of raw materials has issues such as susceptibility to dehydration and shrinkage, and inconsistent gel strength. Consequently, employing specific treatments to enhance the quality of carrageenan represents a significant current research direction. Common methods for carrageenan modification encompass chemical, biological, and physical modification. Numerous researchers globally have observed that alkali treatment facilitates the de-esterification of the sulfate group in the precursor of κ-carrageenan (μ-type carrageenan), thereby enhancing the formation of 3,6-hydrogen bonds [7]. Hillious et al. [8] found that KCl was able to improve the yield and gel properties of carrageenan, and similar findings were found by Bono et al. [9] in their study of KOH treatment of Kirin carrageenan. Consequently, alkali treatment is an essential component of the conventional carrageenan extraction process. Steam explosion (SE) technology, capable of degrading material structures and hastening the extraction of bioactive compounds, distinguishes itself among various physical pretreatment methods due to its notable benefits in terms of energy usage and processing efficiency, and is extensively applied in the valorization of food by-products and the alteration of isolated bioactive substances [10]. Yi et al. [11] reported that SE treatment of hyssop roots resulted in a significant increase in hyssop polysaccharide content and in vitro antioxidant activity. Cheng et al. [12] demonstrated that SE treatment contributed to the release of polyphenols from *adzuki* bean.

κ-carrageenan forms thermoreversible semisolid gels [13], serving as an excellent gelling agent for jelly production. However, κ-carrageenan is frequently combined with xanthan gum and konjac gum in practice to enhance its brittle and weak elasticity, thereby improving the gelation properties of jellies. Therefore, the objective of this study was to evaluate the effect of SE-assisted alkali treatment on the structural and physicochemical properties of κ-carrageenan prepared from *Eucheuma*, and subsequently to apply the treated κ-carrageenan in jelly production, while also investigating the quality characteristics of κ-carrageenan from various preparation methods in jelly applications.

## 2. Results and Discussion

### 2.1. Physicochemical Properties

**Figure 2:**
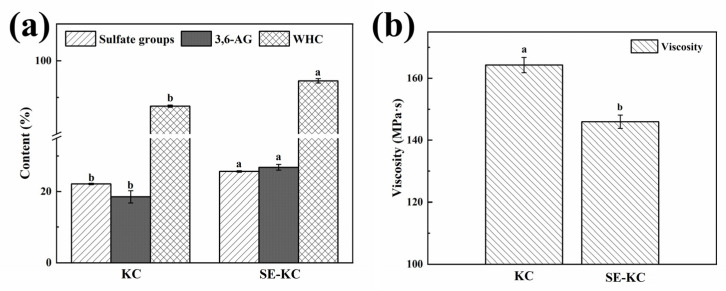
(**a**) Sulfate group content, 3,6-AG content and WHC of KC and SE-KC, (**b**) Viscosity of KC and SE-KC. Values with different letters in the same indicator are significantly different (*p* < 0.05).

Some studies have shown that the number of sulfate groups determines the type of carrageenan [14] and affects biological activities, such as water solubility and antioxidant properties [15]. As shown in Figure 2a, the sulfate content in κ-carrageenan increased by 15.86% following SE pretreatment, potentially due to alterations in the molecular structure of carrageenan during the treatment, which facilitated the exposure of sulfate groups and consequently enhanced the solubility of the κ-carrageenan extracted through SE treatment.

The 3,6-AG content reduces the hydrophilicity of galactose residues, shifts the molecular conformation, and exhibits superior gelling properties, which are positively correlated with gel strength. Figure 2a illustrates that the 3,6-AG content increased by 45.08%, which may be attributed to the fact that SE pretreatment promoted the transformation of 1,4-linked D-galactose-6-sulfate in *Eucheuma* alga into 1,4-linked lactose by eliminating the sulfate group, thereby forming additional 3,6-anhydrous bridges, reducing the hydrophilicity of galactose residues, and promoting the transformation of C-6 sulfate group on the residues of μ-carrageenan (the precursor of κ-carrageenan) into 3,6-endo ether bonds. This change in conformation from ^1^C_4_ to ^4^C_1_ enhances the formation of cross-linkages [5], which are beneficial for the development of a cross-linking network; consequently, it improves the gel properties of carrageenan. This is also the fundamental reason for the significant increase in gel strength by SE treatment.

Viscosity is an important indicator of carrageenan quality. It was found that the viscosity of κ-carrageenan decreased by 11.15% after SE treatment, which may be attributed to the fact that SE treatment modifies the carrageenan, leading to partial unchaining of the molecular chains of κ-carrageenan, a reduction in molecular conformational volume, and the cleavage of some molecular bonds, as well as increased pseudoplasticity at elevated temperatures, all of which can impact the viscosity of κ-carrageenan. A 7.90% enhancement in water retention, relative to the non-SE treatment, could be attributed to the optimization of the molecular structure of carrageenan during SE pretreatment. This process likely increased the number of sulfate and hydroxyl groups, augmenting hydrophilicity and hydrogen bonding, which enhanced the attraction to free water and water retention. Additionally, the formation of additional cross-linking structures through covalent bonding post-modification may have resulted in a gel network structure more amenable to water retention. Furthermore, the increased cross-linking through covalent bonding after modification may render the gel network structure more effective at retaining water molecules, thus mitigating the hydrophobicity enhancement effect induced by the elevated 3,6-AG content [16].

### 2.2. Fourier Transform Infrared Spectroscopy (FT-IR)

**Figure 3:**
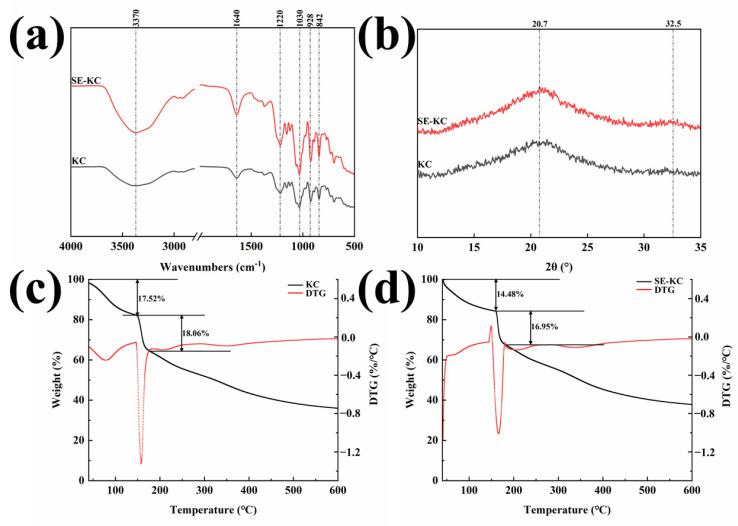
(**a**) FT-IR spectra of KC and SE-KC, (**b**) XRD of KC and SE-KC, (**c**,**d**) Thermogravimetric analysis curves of KC and SE-KC.

Fourier transform infrared spectroscopy was used to characterize the key functional groups of carrageenan samples prepared under different process conditions. As can be seen from Figure 3a, there is a strong broad peak near 3370 cm^−1^, which is the inter- and intramolecular stretching vibration of the hydroxyl group [17]. Notably, the absorption intensity of SE-KC is significantly enhanced, suggesting that the saturated vapor during the SE process intensifies the disruption of hydrogen bonding and liberates a greater number of hydroxyl groups [18]. The absorption peak near 1640 cm^−1^ corresponds to the characteristic stretching vibration of the carbonyl group (C=O), and the increased absorption peak intensity after modification indicates an elevated carbonyl group content in SE-KC. This alteration likely promotes intermolecular interactions, thereby enhancing the water-binding capacity of the polymer [19]. The absorption band observed in the region of 1260–1210 cm^−1^ is indicative of the presence of sulfate groups [20]. Consequently, the absorption peak at 1220 cm^−1^ represents a characteristic vibration of polysaccharide sulfate [21]. The SE-treated κ-carrageenan samples exhibited a more pronounced absorption peak at this wavelength, suggesting an increase in the total sulfate group content due to the SE treatment. The absorption peak at 1030 cm^−1^ corresponds to the stretching vibration of the C-O-C bond in the sugar ring structure [22]. The marked enhancement of the absorption peak of κ-carrageenan post-SE treatment at this wavelength suggests significant alterations in the molecular conformation, along with improved hydrophilicity and stability. The absorption peak near 928 cm^−1^ corresponds to the vibration of 3,6-anhydrogalactoside bond, which corresponds to 3,6-anhydrogalactose and is one of the typical characteristic absorption peaks of κ-carrageenan. Meanwhile, the strong absorption peak near 842 cm^−1^ indicated that the sulfate group was attached to the C_4_ position of the pyran ring, which could accurately determine the position of the sulfate, further confirming that both samples were κ-carrageenan [19]. Comparison of the infrared spectra of κ-carrageenan samples prepared by SE treatment versus the traditional process revealed significantly enhanced absorption peak intensities near 928 cm^−1^ and 842 cm^−1^ in the former, suggesting an increase in sulfate group and 3,6-AG content due to SE treatment, consistent with the findings in Figure 2a. These findings suggest that SE treatment has a minimal impact on the molecular structure and key functional groups of κ-carrageenan, primarily modifying it by breaking glycosidic bonds and reducing the molecular chain length without introducing new groups or compromising the original structural features.

### 2.3. X-Ray Diffraction (XRD)

Figure 3b displays a distinct broad peak with a large area, rounded peak shape and high peak intensity at the position of 2θ about 20°. This phenomenon indicates a small proportion of crystalline regions in κ-carrageenan, which is dominated by an amorphous state [23]. Meanwhile, a subtle diffraction peak was also observed at the 2θ position of approximately 32°. Further analysis revealed that the diffraction peak intensities of KC at these two positions were significantly higher than those of the SE-treated samples, suggesting that the overall crystallinity of the SE-treated κ-carrageenan was reduced, though some degree of crystallinity was preserved. This alteration likely stems from the disruption to the chemical and hydrogen bonds within κ-carrageenan during the SE treatment process, leading to a rearrangement in the molecular order. Meanwhile, the modification process may have influenced both amorphous and crystalline regions to some extent, encouraging the expansion of amorphous areas [24].

### 2.4. Thermogravimetric Analysis (TGA)

Thermogravimetric analysis is a pivotal analytical technique for assessing material mass changes across a temperature gradient. As shown in Figure 3c, d, three distinct stages of mass loss were observed for the κ-carrageenan samples during heat treatment. During the initial stage, the mass loss for the conventionally prepared κ-carrageenan was approximately 17.52%, whereas the SE-prepared sample exhibited a mass loss of 14.48%. This initial mass loss is primarily due to the evaporation of free water molecules. As the temperature increases, free water molecules gain sufficient energy to overcome interaction forces, thereby escaping from the sample. During the second stage, the mass loss of κ-carrageenan prepared by the conventional process was approximately 18.06% within the temperature interval of 150–171 °C, while samples prepared by the SE process experienced a mass loss of about 16.95% within the interval of 160–180 °C. The mass loss at this stage may be associated with the evaporation of bound water. There were strong interactions between bound water molecules and the polysaccharide chains of κ-carrageenan, but these interactions were disrupted at higher temperatures, leading to the release of bound water molecules. Both samples further decomposed at a slow rate during the third stage, with the conventionally prepared κ-carrageenan experiencing a mass loss of approximately 39.09%, and the SE-modified κ-carrageenan exhibiting a mass loss of about 41.90%. This increase in mass loss can be ascribed to the continuous degradation of κ-carrageenan at elevated temperatures, characterized by numerous breaks in the polysaccharide chains [21]. The SE treatment likely enhanced the binding capacity of κ-carrageenan with water molecules by introducing new functional groups or altering intermolecular interactions, thereby increasing the content of bound water and improving the overall thermal stability of the material. In addition, it is observed that the SE-modified κ-carrageenan commenced significant decomposition at higher temperatures than the conventionally extracted κ-carrageenan, indicating enhanced thermal stability.

In summary, the SE treatment enhances the thermal stability of κ-carrageenan by modifying its molecular structure and interactions with water molecules, offering a theoretical foundation for the evaluation and optimization of the performance of κ-carrageenan in high-temperature application environments.

### 2.5. Low-Field Nuclear Magnetic Resonance (LF-NMR)

**Figure 4:**
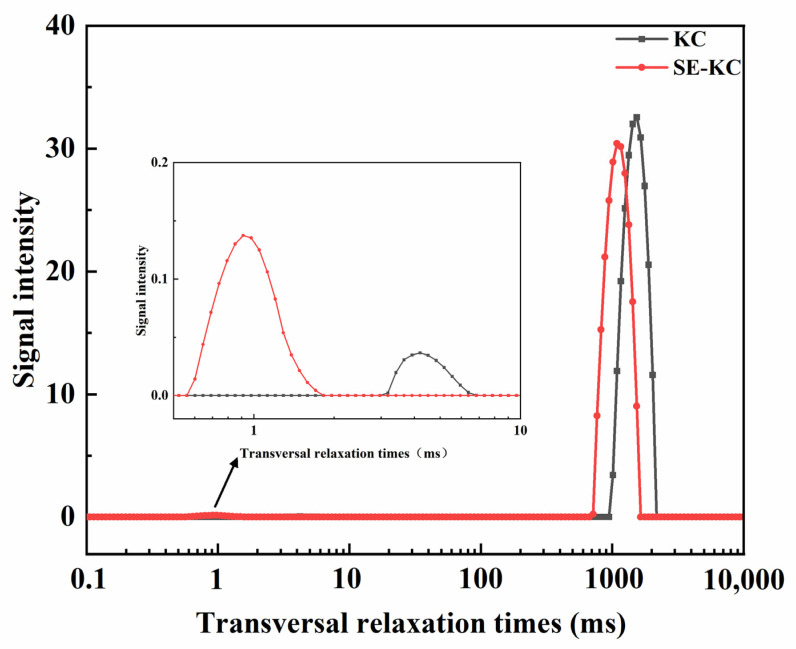
Effects of different preparation processes on the water distribution of κ-carrageenan gel.

Low-field nuclear magnetic resonance is extensively utilized in the field of food science, particularly for the rapid detection of foodstuffs, owing to its rapid and non-destructive characteristics. The distinct peaks in the T_2_ relaxation curve at 0.1–10 ms (T_21_), 10–100 ms (T_22_), and 100–1000 ms (T_23_) correspond to bound water, semi-bound water, and free water, respectively [25]. As shown in Figure 4 and Table 1, all gel systems in the LF-NMR analysis exhibited two distinct relaxation peaks. T_21_ was located at 0.56–7.32 ms and T_22_ at 666.99–2171.12 ms, indicating that each gel system contained both bound water and free water. Bound water, typically found between 0.1–10 ms, forms stronger chemical bonds with solute molecules, resulting in a faster relaxation rate and shorter relaxation time, corresponding to T_21_, while free water, being less restricted, has a longer relaxation time, corresponding to the T_22_ peak. Further analysis indicates that the relaxation peak in the SE-modified samples shifted toward a shorter relaxation time; this shift suggests a transition in the state of water molecules from free to bound, likely due to the SE process disrupting numerous hydrogen bonds and rearranging molecular chains [18], thereby altering κ-carrageenan’s interaction with water and promoting the formation of additional hydrogen bonds to enhance its binding capacity with water. Table 1 indicates that the proportion of bound water increased after modification, and this trend suggests that the SE treatment effectively enhanced the water-holding capacity of κ-carrageenan gels. This is corroborated by the observed increase in the intensity of hydroxyl group absorption peaks. The SE treatment is crucial for optimizing water molecule distribution and enhancing the functionality of the gel system.

### 2.6. Texture Profile Analysis (TPA)

Texture profile analysis is an experimental method that emulates human mastication and is designed to simulate the processing behavior of food in the oral cavity to investigate the textural properties of food gels and their performance following structural disruption. Table 2 displays the effect of κ-carrageenan addition on the textural properties of jellies prepared by two different processes. The study found that the textural attributes of the jelly were improved by incorporating an appropriate amount of κ-carrageenan, and the hardness, elasticity, and gelatinization of SE-treated κ-carrageenan were significantly increased (*p* < 0.05). Cohesiveness indicates the internal cohesion of the gel network and the structural integrity of the gel, and changes in the gel’s backbone structure generally influence cohesiveness [26]. Table 2 shows that cohesiveness does not significantly change with increased carrageenan addition, yet the SE-treated jellies exhibit greater cohesiveness, suggesting that SE pretreatment enhances the internal strength of the gel network. Shen et al. [27] also report that carrageenan concentration has a minimal impact on cohesiveness. Optimal springiness was achieved at a carrageenan addition level of 1.5%. Comparing the springiness data between the two groups revealed that the springiness of the jellies in the SE process group was consistently better than that of the traditional process group, further validating that SE treatment enhances the cohesiveness of κ-carrageenan, thereby improving jelly performance. This indicates that the SE process significantly improves the gel properties of κ-carrageenan and enhances the textural properties of κ-carrageenan in food applications.

### 2.7. Gel Strength of Jelly

**Figure 5:**
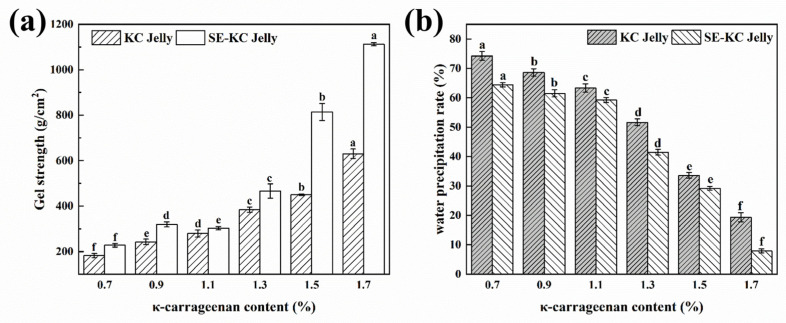
(**a**) Influence of the addition amount of carrageenan prepared by the two processes on the gel strength of jelly, (**b**) Influence of the addition amount of carrageenan prepared by the two processes on the freezing and thawing stability of jelly. Values with different letters in the same process differ significantly (*p* < 0.05).

Gel strength indicates the degree of gel network formation and compactness and can be used to assess the impact of processing and handling methods on food product quality. Figure 5a shows that the SE process can significantly increase the gel strength of κ-carrageenan. For κ-carrageenan concentrations of 0.7–1.3%, the difference in gel strength between the two preparation methods was minimal. At the mass fraction exceeding 1.3%, the gel strength of jellies prepared by the SE group was markedly enhanced. Relative to the traditional process, the gel strength of κ-carrageenan increased significantly by 80.9% at an addition level of 1.5%. The gel strength of jelly in the SE group was enhanced by 76.54% at an addition level of 1.7%, suggesting that increasing the proportion of κ-carrageenan significantly influences the gel strength, promoting the formation of a κ-carrageenan-dominated reticulation [28], especially at higher κ-carrageenan concentrations. This further demonstrates that the SE process has a positive effect on improving the gel properties of κ-carrageenan.

### 2.8. Freezing and Thawing Stability of Jelly

Freezing and thawing stability has a critical impact on the quality and safety of frozen foods. Temperature fluctuations during cold chain storage can impact the gel matrix within food products, triggering moisture migration and phase changes in water. These alterations can induce dehydration and shrinkage of the gel matrix, thereby compromising the structural integrity and sensory attributes of the food product [13]. Freeze–thaw stability in a sample is indicated the water precipitation rate; the lower the rate is, the greater is the freeze–thaw stability [26]. Figure 5b shows that the water precipitation rates of both jelly samples decreased as the mass fraction of κ-carrageenan increased. This result may be related to the fact that the addition of κ-carrageenan increased the proportion of bound water in the gel system, which resulted in a more effective inhibition of ice crystal formation during the freeze–thaw cycle and a significant enhancement of the gel’s resistance to mechanical stress. The κ-carrageenan jellies prepared by the SE process showed lower water precipitation rates at all mass fraction levels than those prepared by the conventional process, suggesting that the freeze–thaw stability of the modified κ-carrageenan was enhanced and its binding with water molecules could be better maintained during the freezing process. At a κ-carrageenan addition level of 1.7%, the water precipitation rate of the jelly reached the lowest value (7.96 ± 0.69%), a reduction of 58.82% compared to the jelly from the traditional process group (19.34 ± 1.61%) at the same mass fraction, indicating that the freeze–thaw stability of the jelly was significantly improved at this mass fraction. This enhancement may be attributed to alterations in the molecular conformation of κ-carrageenan following the SE treatment, and an increase in the bound water content within the system, leading to the formation of a gel network with enhanced structural stability. This structural optimization effectively prevented ice crystal formation during the freeze–thaw cycle and minimized water loss, significantly improving the freeze–thaw stability of the jelly.

## 3. Conclusions

The results show that the preparation of κ-carrageenan by SE pretreatment combined with alkali treatment had a positive effect on the enhancement of the quality characteristics of the jellies. SE is an environmentally friendly and innovative pretreatment method, whereby the cell wall of *Eucheuma* raw material is disrupted through a process of high-temperature cooking and instantaneous decompression, facilitating the release of gelatin, and increasing the content of sulfate groups and 3,6-AG. Among them, 3,6-AG is a characteristic group of κ-carrageenan, which is positively correlated with the gel properties of carrageenan. Overall gel properties and physicochemical properties were enhanced. SE treatment optimized the molecular structure of the gel, resulting in jellies with improved gel strength, elasticity, and adhesion, along with greater resistance to deformation, thus positively impacting the shape retention of the jellies. In conclusion, the SE-assisted alkali treatment process significantly improves the performance of κ-carrageenan in jelly and other food applications. This study provides a theoretical basis for exploring SE as an auxiliary treatment method to improve the physicochemical properties of κ-carrageenan.

## 4. Materials and Methods

### 4.1. Materials

*Eucheuma* was provided by the Fujian Lukki Food Colloid Co., Ltd. (Zhangzhou, China). NaOH and KCl, AR, purchased from the Shanghai McLean Biochemical Technology Co., Ltd. (Shanghai, China); industrial alcohol, 95%, was purchased from the Guangtong Chemical Co., Ltd. (Fuzhou, China). Peach juice was provided by the Guangzhou Unity Enterprise Co. (Guangzhou, China).

### 4.2. SE-Assisted Alkali Extraction of κ-Carrageenan from Eucheuma

Based on the preliminary experiments conducted in this study, *Eucheuma* was washed and drained until there was no obvious water droplet and was put into the silo with an 80% charge ratio. The valve was securely tightened, and the material was maintained at an SE pressure of 0.3 MPa for 90 s before being rapidly depressurized. Subsequently, the *Eucheuma* after the collection of bursts was put into the oven at 60 °C to dry for spare parts.

A specific weight of *Eucheuma* was measured before and after SE, then treated with a 10% KOH solution at 85 °C in a water bath for 3.5 h, washed until the pH reached 7–8, and subsequently heated in a water bath at 80 °C for 2 h, and then centrifuged at 3580× *g* for 15 min to separate the algal residue from the gelatinous liquid. The centrifuged gel solution was cooled to room temperature (25 °C) with constant stirring, then mixed with three times the volume of alcohol, and continuously stirred with a glass rod to allow the gel bars to precipitate, and then left to stand for 12 h. The gel bars precipitated from the alcohol were extruded from the alcohol by means of a 200-mesh filter cloth, and then placed in an oven at 60 °C for 24 h after drying and milling to obtain the finished product κ-carrageenan. The κ-carrageenan extracted from non-SE-treated *Eucheuma* is denoted as KC, and the κ-carrageenan extracted from SE-treated *Eucheuma* is denoted as SE-KC.

### 4.3. Jelly-Making Process

A specific mass of the two previously prepared κ-carrageenan samples was weighed and mixed with peach juice solution (containing 0.2% KCl), heated in a water bath at 80 °C, and stirred magnetically for 30 min until the κ-carrageenan was completely dissolved in the peach juice solution. We then used a 100-mesh food-grade stainless steel sieve mesh filtration to remove part of the colloidal mass and residue that is not mixed uniformly; the homogeneous solution was poured into a silicone mold at 25 °C cooling molding; solidification of the molded jelly demolding was placed in a prepared transparent plastic box at 4 °C storage. The jelly gels were formulated with varying κ-carrageenan mass fractions of 0.7%, 0.9%, 1.1%, 1.3%, 1.5% and 1.7%.

### 4.4. Physicochemical Properties of κ-Carrageenan

#### 4.4.1. Sulfate Content

**Figure 6:**
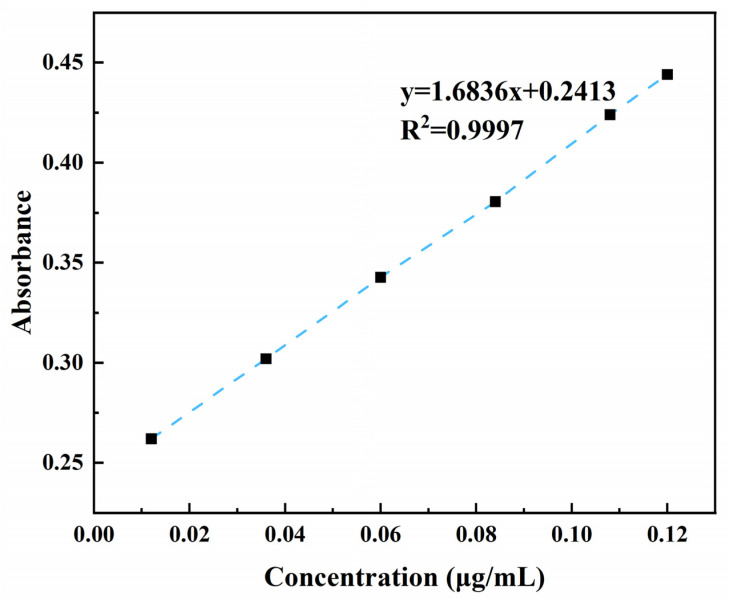
Standard curve for sulfate content.

The sulfate content of κ-carrageenan was quantified using the turbidimetric method with BaSO_4_ [29]. Briefly, K_2_SO_4_ crystals were dried to constant weight and precisely weighed to 108.75 mg, then dissolved in 1 mol/L hydrochloric acid to prepare a 0.6 mg/mL sulfuric acid-based standard solution. A range of standard solutions with varying concentrations were prepared by pipetting 0.02 mL to 0.20 mL of the sulfate-based standard solution and diluting to 0.2 mL with hydrochloric acid. A total of 0.20 mL of hydrochloric acid solution was added to the blank control; we added 3.8 mL of trichloroacetic acid and 1.0 mL of barium chloride solution, followed by incubation at room temperature (25 °C) for 15 min after thorough mixing. The absorbance A_1_ was measured at 360 nm using a UV spectrophotometer (EVOLUTION Pro, Thermo Fisher Scientific, Waltham, MA, USA), while the absorbance A_2_ was determined for the sample containing gelatin in place of barium chloride. The standard curve was constructed based on the absorbance difference (A_1_–A_2_) versus the sulfate concentration, and the sulfate content was calculated from this curve. The standard curve is shown in Figure 6.

#### 4.4.2. 3,6-AG Content

The 3,6-AG content was determined with reference to the method of Yaphe [30] with slight modifications. Briefly, 50 mg of the κ-carrageenan sample was weighed, dissolved by heating, and cooled to room temperature (25 °C) and the volume was adjusted to a fixed mark. A 2 mL aliquot of the sample solution was transferred to a test tube, followed by the addition of 15 mL of color developer, and the mixture was reacted at 80 °C for 10 min, then immediately cooled in an ice bath. The absorbance value A was measured by a spectrophotometer (EVOLUTION Pro, Thermo Fisher Scientific, USA). Meanwhile, a colorimetric analysis was conducted using a fructose standard solution at 80 μg/mL as a proxy for 3,6-AG. Chromogen A was prepared by dissolving 130 mg of resorcinol in 100 mL of anhydrous ethanol and stored under conditions of low temperature and protected from light; chromogen B consisted of a 12 M hydrochloric acid solution. Prior to colorimetry, chromogen A and chromogen B were mixed at a ratio of 1:10.

The formula for the calculation of the 3,6-AG content was as follows:

$$C(\frac{\mu g}{\mathrm{mL}})=\frac{A}{A_{0}}\times C_{0}\times 144\times 1.087$$

where: C represents the concentration of 3,6-AG in the sample (μg/mL), A represents the absorbance value of the sample measured by colorimetry, A_0_ represents the absorbance value of the fructose measured by colorimetry, C_0_ represents the molar concentration of fructose (μmol/mL), 144 is the molecular weight of 3,6-AG, and 1.087 is the correction factor of the fructose used as the standard.

#### 4.4.3. Viscosity

The viscosity of the κ-carrageenan solution was measured using an SNB-2 digital viscometer (Shanghai Tianmei Balance Instrument Co., Ltd., Shanghai, China). Briefly, to prepare the 1.5% κ-carrageenan solution, the sample was fully diffused and then heated with an electromagnetic stirring heater until dissolved, and the water evaporated during the heating process was replenished after the sample was completely dissolved. The viscosity measurement was conducted in a thermostatically controlled water bath set to 75 °C, employing a #1 rotor at 30 r/min [9].

#### 4.4.4. Water-Holding Capacity (WHC)

The 1.5% κ-carrageenan solution was prepared and allowed to gel, followed by centrifugation of the gel samples at 3580× *g* for 15 min [31]. Subsequently, the excess water was eliminated and the WHC was calculated according to the following formula:

$$\mathrm{WHC}(\%)=\frac{m_{1}}{m_{0}}\times 100$$

where: m_0_ and m_1_ are the weight of the gel sample before and after centrifugation.

### 4.5. FT-IR

Fourier transform infrared spectroscopy (Thermo Scientific, USA) was utilized to examine the alterations in the functional groups of κ-carrageenan before and after the modification process. Briefly, the samples were prepared by mixing with KBr and compressing with a tablet press, followed by 32 consecutive scans in the spectral range of 4000–500 cm^−1^ at a resolution of 4 cm^−1^ [32].

### 4.6. XRD

X-ray diffraction (Shimadzu Corporation, Kyoto, Japan) was employed to characterize the crystal structure of the samples. The XRD patterns were recorded with a sampling time of 0.15 s, an accelerating voltage of 40 kV, and an applied current of 30 mA over a 2θ range of 10–90° [2].

### 4.7. TGA

The thermal stability of κ-carrageenan before and after SE treatment was assessed using a thermogravimetric analyzer (TGA8000, PerkinElmer, Waltham, MA, USA). We weighed 3–6 mg of the κ-carrageenan sample powder in a crucible, and the sample was subjected to a temperature ramp from 40 °C to 600 °C at a heating rate of 10 °C/min in a nitrogen atmosphere with a purge gas flow rate of 20 mL/min, while we monitored the mass change and the first derivative of the mass loss rate (DTG) [31].

### 4.8. LF-NMR

The 1.5% κ-carrageenan gel samples (containing 0.2% KCl) were prepared and allowed to set at room temperature. The moisture distribution of the carrageenan gel samples was assessed using an NMI 20-060 H-I instrument (Shanghai Niumag Co., Ltd., Shanghai, China), employing the methodology described by Wang [33]. Briefly, a sample weighing approximately 8 g was prepared and examined within a 60 mm NMR tube. The parameters for the Carr–Purcell–Meiboom–Gill (CPMG) sequence were configured as: 90° pulse width P1 = 14.52 μs, 180° pulse width P2 = 28.48 μs, sampling frequency = 200 kHz, echo time = 0.5 ms, number of echoes = 20,000, inter-pulse delay = 1500 ms, and number of scans = 8. The Simultaneous Iterative Reconstruction Technique (SIRT) was applied for data processing. During this process, the data points and relaxation time points were established at 200 each, with a filter setting of 3, and a relaxation time range of 0.01–10,000 ms.

### 4.9. Texture Profile Analysis

The texture profile analysis of jellies was determined according to the method of Jiang [32] with a texture meter (TMS-Pilot; Beijing Yingsheng Hengtai Technology Co., Ltd., Beijing, China). Briefly, a disc extrusion probe with a diameter of 75 mm was used; the speed of pre-testing and testing was 60 mm/min, the speed of post-testing was 10 mm/s, the degree of compression was 40%, the trigger force was 0.75 N, the interval between the two compression times was 5 s, and the trigger type was automatic. The resulting parameters, including hardness, cohesiveness, stringiness, and gumminess, were measured using the texture analyzer.

### 4.10. Gel Strength

The gel strength of the jellies was determined using a TMS-PILOT texture analyzer. A 1.5% carrageenan solution (containing 0.2% KCl) was prepared, heated in a water bath until the carrageenan was fully dissolved, and then allowed to set at room temperature for 10 h before assessing the gel strength using a texture analyzer. The initial force was set to 0.75 N, and the deformation distance was 15 mm [9].

### 4.11. Freezing and Thawing Stability

The freezing and thawing stability of the jellies was evaluated using a modified approach based on the method described by Zhang [34]. Briefly, jelly samples prepared with 1.5% κ-carrageenan (containing 0.2% KCl) were frozen at −20 °C for 24 h, then thawed at 25 °C for 6 h and centrifuged at 3580× *g* for 20 min; the water precipitation rate was calculated as the difference in water loss before and after centrifugation. The precipitation rate (%) of the jelly was calculated according to the following formula.

$$\mathrm{water}\;\mathrm{precipitation}\;\mathrm{rate}\left(\%\right)=\frac{W_{1}-W_{2}}{W_{1}}\times 100$$

where W_1_ and W_2_ are the weight of the gel before and after centrifugation.

### 4.12. Statistical Analysis

Three replications of each set of experiments were performed and the results were presented as mean ± standard deviation. Data analysis was performed using Excel and SPSS 26, with graphical representation performed in Origin2018.

## Figures and Tables

**Table 1:** Effects of different preparation processes on the water distribution of κ-carrageenan gel.

	KC		SE-KC	
	T_21_	T_22_	T_21_	T_22_
peak onset time/ms	2.97	943.79	0.56	666.99
peak point time/ms	4.20	1534.37	0.91	1084.37
peak end time/ms	7.32	2171.12	1.83	1644.67
peak ratio (%)	0.10	99.90	0.49	99.51

**Table 2:** Effect of κ-carrageenan addition on textural properties of jellies by conventional and SE processes.

	Carrageenan Content/%	Hardness (N)	Cohesiveness (%)	Springiness (mm)	Gumminess (N)
KC Jelly	0.7	20.87 ± 2.91 ^e^	0.4 ± 0.1 ^a^	2.29 ± 0.09 ^c^	7.93 ± 2.12 ^d^
	0.9	31.01 ± 3.32 ^d^	0.33 ± 0.15 ^a^	2.57 ± 0.21 ^ab^	11.44 ± 5.94 ^d^
	1.1	43.45 ± 7.05 ^c^	0.5 ± 0.1 ^a^	2.8 ± 0.05 ^a^	19.89 ± 2.59 ^c^
	1.3	59.68 ± 1.24 ^b^	0.5 ± 0 ^a^	2.78 ± 0.08 ^a^	30.03 ± 0.96 ^b^
	1.5	70.19 ± 4.17 ^a^	0.5 ± 0 ^a^	2.69 ± 0.05 ^a^	34.18 ± 1.25 ^bc^
	1.7	74.76 ± 4.77 ^a^	0.5 ± 0 ^a^	2.37 ± 0.17 ^bc^	36.85 ± 1.28 ^a^
SE-KC Jelly	0.7	24.19 ± 0.5 ^e^	0.47 ± 0.01 ^c^	2.61 ± 0.07 ^d^	11.32 ± 0.35 ^d^
	0.9	31.13 ± 1.11 ^d^	0.51 ± 0.03 ^bc^	2.9 ± 0.16 ^c^	15.77 ± 0.77 ^cd^
	1.1	41.7 ± 1.69 ^c^	0.51 ± 0.03 ^a^	3.19 ± 0.08 ^ab^	21.37 ± 0.48 ^c^
	1.3	55.23 ± 3.89 ^b^	0.53 ± 0.02 ^a^	3.03 ± 0.23 ^abc^	29.14 ± 1.13 ^b^
	1.5	67.09 ± 2.15 ^a^	0.52 ± 0.01 ^ab^	3.27 ± 0.07 ^a^	35.09 ± 1.34 ^a^
	1.7	69.05 ± 4.05 ^a^	0.47 ± 0.08 ^a^	2.94 ± 0.13 ^bc^	32.19 ± 6.95 ^ab^

Note: Data are expressed as standard deviation ± mean, and different superscript letters in the same column of data indicate significant differences (*p* < 0.05).

## Data Availability

Data are contained within the article.

## References

[1] Zheng L.-X., Chen X.-Q., Cheong K.-L. (2020). Current Trends in Marine Algae Polysaccharides: The Digestive Tract, Microbial Catabolism, and Prebiotic Potential. Int. J. Biol. Macromol..

[2] Jumaidin R., Sapuan S.M., Jawaid M., Ishak M.R., Sahari J. (2017). Characteristics of Eucheuma Cottonii Waste from East Malaysia: Physical, Thermal and Chemical Composition. Eur. J. Phycol..

[3] Cao C., Feng Y., Kong B., Xia X., Liu M., Chen J., Zhang F., Liu Q. (2021). Textural and Gel Properties of Frankfurters as Influenced by Various *κ*-Carrageenan Incorporation Methods. Meat Sci..

[4] Brenner T., Tuvikene R., Fang Y., Matsukawa S., Nishinari K. (2015). Rheology of Highly Elastic Iota-Carrageenan/Kappa-Carrageenan/Xanthan/Konjac Glucomannan Gels. Food Hydrocoll..

[5] Campo V.L., Kawano D.F., da Silva D.B., Carvalho I. (2009). Carrageenans: Biological Properties, Chemical Modifications and Structural Analysis—A Review. Carbohydr. Polym..

[6] Udo T., Mummaleti G., Mohan A., Singh R.K., Kong F. (2023). Current and Emerging Applications of Carrageenan in the Food Industry. Food Res. Int..

[7] Nurani W., Anwar Y., Batubara I., Arung E.T., Fatriasari W. (2024). *Kappaphycus Alvarezii* as a Renewable Source of Kappa-Carrageenan and Other Cosmetic Ingredients. Int. J. Biol. Macromol..

[8] Hilliou L., Larotonda F.D.S., Abreu P., Ramos A.M., Sereno A.M., Gonçalves M.P. (2006). Effect of Extraction Parameters on the Chemical Structure and Gel Properties of κ/ι-Hybrid Carrageenans Obtained from *Mastocarpus Stellatus*. Biomol. Eng..

[9] Bono A., Anisuzzaman S.M., Ding O.W. (2014). Effect of Process Conditions on the Gel Viscosity and Gel Strength of Semi-Refined Carrageenan (SRC) Produced from Seaweed (*Kappaphycus Alvarezii*). J. King Saud Univ.—Eng. Sci..

[10] Wang C., Lin M., Yang Q., Fu C., Guo Z. (2023). The Principle of Steam Explosion Technology and Its Application in Food Processing By-Products. Foods.

[11] Yi J., Li X., Wang S., Wu T., Liu P. (2022). Steam Explosion Pretreatment of *Achyranthis Bidentatae* Radix: Modified Polysaccharide and Its Antioxidant Activities. Food Chem..

[12] Cheng A., Hou C., Sun J., Wan F. (2020). Effect of Steam Explosion on Phenolic Compounds and Antioxidant Capacity in Adzuki Beans. J Sci Food Agric.

[13] Wang Y., Yuan C., Cui B., Liu Y. (2018). Influence of Cations on Texture, Compressive Elastic Modulus, Sol-Gel Transition and Freeze-Thaw Properties of Kappa-Carrageenan Gel. Carbohydr. Polym..

[14] Diharmi A., Fardiaz D., Andarwulan N., Heruwati E.S. (2017). Chemical and Physical Characteristics of Carrageenan Extracted from Eucheuma Spinosum Harvested from Three Different Indonesian Coastal Sea Regions. Phycol. Res..

[15] Rudke A.R., de Andrade C.J., Ferreira S.R.S. (2020). *Kappaphycus Alvarezii* Macroalgae: An Unexplored and Valuable Biomass for Green Biorefinery Conversion. Trends Food Sci. Technol..

[16] Xu X., Jiang F., Lin K., Fang J., Chen F., Ru Y., Weng H., Xiao Q., Yang Q., Xiao A. (2024). Anhydride Esterification to Regulate Water Migration and Reduce Ice Crystal Formation in κ-Carrageenan Gel during Freezing. Food Hydrocoll..

[17] Paula G.A., Benevides N.M.B., Cunha A.P., de Oliveira A.V., Pinto A.M.B., Morais J.P.S., Azeredo H.M.C. (2015). Effect of K-Carrageenan on the Gelation Properties of Oyster Protein. Food Hydrocoll..

[18] Wan F., Feng C., Luo K., Cui W., Xia Z., Cheng A. (2022). Effect of Steam Explosion on Phenolics and Antioxidant Activity in Plants: A Review. Trends Food Sci. Technol..

[19] Şen M., Erboz E.N. (2010). Determination of Critical Gelation Conditions of κ-Carrageenan by Viscosimetric and FT-IR Analyses. Food Res. Int..

[20] Gómez-Ordóñez E., Rupérez P. (2011). FTIR-ATR Spectroscopy as a Tool for Polysaccharide Identification in Edible Brown and Red Seaweeds. Food Hydrocoll..

[21] Tye Y.Y., HPS A.K., Kok C.Y., Saurabh C.K. (2018). Preparation and Characterization of Modified and Unmodified Carrageenan Based Films. IOP Conf. Ser. Mater. Sci. Eng..

[22] Li Z., Cheong K.-L., Song B., Yin H., Li Q., Chen J., Wang Z., Xu B., Zhong S. (2024). Preparation of κ-Carrageenan Oligosaccharides by Photocatalytic Degradation: Structural Characterization and Antioxidant Activity. Food Chem. X.

[23] Xi H., Wang A., Qin W., Nie M., Chen Z., He Y., Wang L., Liu L., Huang Y., Wang F. (2023). The Structural and Functional Properties of Dietary Fibre Extracts Obtained from Highland Barley Bran through Different Steam Explosion-Assisted Treatments. Food Chem..

[24] Ouyang H., Guo B., Hu Y., Li L., Jiang Z., Li Q., Ni H., Li Z., Zheng M. (2023). Effect of Ultra-High Pressure Treatment on Structural and Functional Properties of Dietary Fiber from Pomelo Fruitlets. Food Biosci..

[25] Jiang S., Ma Y., Wang Y., Wang R., Zeng M. (2022). Effect of κ-Carrageenan on the Gelation Properties of Oyster Protein. Food Chem..

[26] Lyu M., Lyu J., Wang F., Xie J., Bai L., Bi J. (2023). Analysis of Gelation Properties of Peach-κ-Carrageenan Gels: Effect of Erythritol. Bioact. Carbohydr. Diet. Fibre.

[27] Shen Y.-R., Kuo M.-I. (2017). Effects of Different Carrageenan Types on the Rheological and Water-Holding Properties of Tofu. LWT.

[28] Voron’ko N.G., Derkach S.R., Vovk M.A., Tolstoy P.M. (2017). Complexation of κ-Carrageenan with Gelatin in the Aqueous Phase Analysed by 1H NMR Kinetics and Relaxation. Carbohydr. Polym..

[29] Verma B.C., Swaminathan K., Sud K.C. (1977). An Improved Turbidimetric Procedure for the Determination of Sulphate in Plants and Soils. Talanta.

[30] Yaphe W., Arsenault G.P. (1965). Improved Resorcinol Reagent for the Determination of Fructose, and of 3,6-Anhydrogalactose in Polysaccharides. Anal. Biochem..

[31] Wang C., Lin M., Li Y., Guo Z. (2024). Improvement of Soluble Dietary Fiber Quality in *Tremella Fuciformis* Stem by Steam Explosion Technology: An Evaluation of Structure and Function. Food Chem..

[32] Jiang F., Liu Y., Xiao Q., Chen F., Weng H., Chen J., Zhang Y., Xiao A. (2022). Eco-Friendly Extraction, Structure, and Gel Properties of ι-Carrageenan Extracted Using Ca(OH)_2_. Mar. Drugs.

[33] Wang J., Ding Y., Wang M., Cui T., Peng Z., Cheng J. (2021). Moisture Distribution and Structural Properties of Frozen Cooked Noodles with NaCl and Kansui. Foods.

[34] Zhang L., Xiao Q., Zhang Y., Weng H., Wang S., Chen F., Xiao A. (2023). A Comparative Study on the Gel Transition, Structural Changes, and Emulsifying Properties of Anhydride-Esterified Agar with Varied Degrees of Substitution and Carbon Chain Lengths. Food Hydrocoll..

